# Effects of prolonged working hours on heart rate variability in internal medicine physicians

**DOI:** 10.1038/s41598-022-23538-6

**Published:** 2022-11-03

**Authors:** Teerapat Nantsupawat, Pongsatorn Tungsuk, Siriluck Gunaparn, Arintaya Phrommintikul, Wanwarang Wongcharoen

**Affiliations:** Division of Cardiology, Department of Internal Medicine, Faculty of Medicine, Chiang Mai University, Chiang Mai, 50200 Thailand

**Keywords:** Cardiology, Health care

## Abstract

Prior studies have utilized heart rate variability (HRV) as the assessment tools for psychological and physiological stress during 24-h shift. However, data regarding effects of prolonged working hours > 24 h on HRV are limited. We aimed to compare between pre- and post-call HRV among physicians who worked 24 plus 8 h. The study included 60 physicians in the internal medicine training. All subjects underwent Holter ECG monitoring for HRV assessment. We compared between HRV of an 8-h regular workday (8am to 4 pm) before on-call duty (pre-call HRV) and an 8-h workday after 24-h on-call duty (post-call HRV). The mean age was 26 ± 2.5 years. Mean total sleep time during on-call duty was 238.9 ± 88.3 min. In overall population, the time-domain and frequency-domain HRV parameters were not different between pre- and post-call day. However, the physicians reported their sleep time in the 1st quartile (< 180 min) had significant increase in SDNN, pNN50, high frequency (HF), and decrease in low/high frequency ratio (LF/HF). In contrast, the physicians reported their sleep time in the 4th quartile (> 307.5 min) had significant decrease in pNN50, LF, HF, and increase in heart rate. Multiple linear regression revealed total sleep time as an independent factor associated with pre- and post-call HRV alterations. More sleep during on call (> 5 h) was associated with HRV pattern suggesting both increased sympathetic activity and reduced parasympathetic activity, while less sleep (< 3 h) during on call was associated with post-call parasympathetic rebound HRV pattern.

## Introduction

Prolonged working over 24 h is not uncommon among physicians. This practice could lead to sleep deprivation, emotional, and physical stress which have been shown to affect work performance and increase rate of medical errors^1^. Not only undermining patient’s safety, prolonged shift work can disturb sleep–wake cycle, eating patterns, social life, and increase risk of ischemic heart disease^2^. There were many methods used to assess psychological stress at work, such as visual analog scale^3^, and Job Content Questionnaire^4^. Searching for the proper assessment tools to measure psychological and physiological stress at work may aid understanding, and facilitate research into stress reduction interventions which may improve short- and/or long-term outcomes for both patients and physicians.

Heart rate variability (HRV) is the fluctuation in the time intervals between adjacent heartbeats. HRV reflects regulation of autonomic balance, cardiovascular, central nervous, endocrine, and respiratory systems, circadian rhythm, baroreceptors, and chemoreceptors^5^. A healthy heart has beat-to-beat variations which indicates the flexibility in adapting to environmental or biopsychosocial challenges. HRV can be measured in ultra-short-term (< 5 min), short-term (< 24 h), and 24-h analysis and can be described as time-domain, frequency-domain, and non-linear measurements. Studies have been shown that low HRV is associated with high job strain^6^, night shift^7^, or 24-h shift^8,9^, and is also a marker of increased cardiovascular risks^10,11^.

Most of the data among physicians and health personnel were limited to HRV monitored during 24-h shift or less. The data regarding HRV during prolonged working hours beyond 24 h are scarce. Therefore, we aimed to measure HRV in physicians who were the internal medicine training during extended working hours beyond 24-h call, as compared to their regular 8-h working day. In addition, we sought to determine the potential factors associated with the HRV alterations in this population. The prevalence of cardiac arrhythmias during normal working hours and after prolonged working hours was also examined.

## Methods

### Study design and participants

This is a cross-sectional study. The volunteered internal medicine residents, interns, externs (age 18–35 years) who had 32-h working periods in Maharaj Nakorn Chiang Mai Hospital, Chiang Mai, Thailand, were enrolled in the study during a period from June 2020 to June 2022. We excluded the participants who used alcohol, caffeine, stimulant drug, or tobacco within 8 h prior to the enrollment. Those with underlying diseases were also excluded.

### Data collection

**Figure 1:**
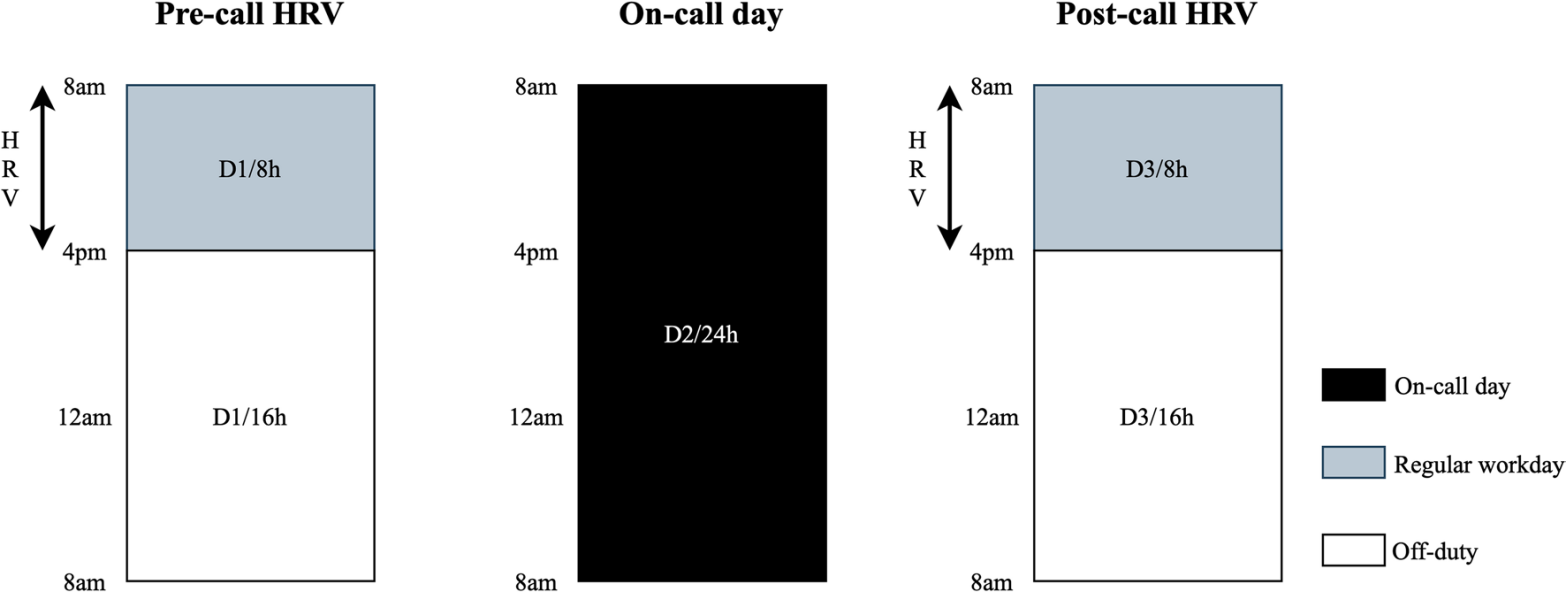
Flow of study comparing 8-h HRV on the day before and after on-call duty.

All subjects underwent Holter ECG monitoring (24-h Holter GE Seer Light Extend, GE Medical Systems, Suzuken Company, Limited, Nagoya, Aichi, Japan) for HRV assessment using MARS® Holter Analysis Workstation. In the internal medicine training, a regular workday starts at 8am to 4 pm. An on-call duty starts at 4 pm to 8am the next morning. At the end of on-call duty, the doctors must continue a regular workday from 8 am to 4 pm. Therefore, on the day of on-call duty, the doctors are compelled to continuously work for a total of 32 h (8am to 4 pm on the next day). In the present study, we compared between the HRV of an 8-h regular workday (8am to 4 pm) before on-call duty (pre-call HRV) and the 8-h workday after 24-h on-call duty (post-call HRV) as shown in Fig. 1. Clinical data were utilized, including age, gender, duration of working hours and duration of sleeping hours.

### Definitions

#### Heart rate variability

Time-domain and frequency-domain analyses were performed according to the standard guidelines. Time-domain HRV indexes were analyzed using statistical methods. The square root of the mean squared differences of successive normal-to-normal (NN) intervals (RMSSD), the standard deviation (SD) of all NN intervals (SDNN), the SD of the average NN intervals calculated over 5-min periods of the entire recording (SDANN), and the proportion of adjacent NN intervals differing by > 50 ms (pNN50) were measured.

Frequency-domain HRV were analyzed using autoregressive power spectral analysis applied to the RR interval time series. The following spectral bands were identified: low frequency (LF) (0.04–0.15 Hz), and high frequency (HF) (0.15–0.4 Hz). Total power (0–0.5 Hz) and the areas below each peak was calculated in absolute units (ms^2^). Total power (TP) is the sum of the energy in the very low frequency (VLF), LF, and HF bands for HRV recording less than 24 h, and represents the global autonomic activity^12^.

The present study was registered at thaiclinicaltrials.org and the identification number is TCTR20210903003. This study was approved by the Faculty of Medicine Ethical Committee with the approval number 268/2564. The investigation was performed according to the Declaration of Helsinki. Informed consent was obtained from all participants.

### Statistical analysis

#### Sample size calculation

We estimated the sample size using the estimation of a two-sample paired-means test to compare the difference of HRV parameters using SDNN during 8-h before on-call duty and during 8-h after on-call duty. The assumption is based on a previous study by Borchini and colleagues^13^ which found that the prolonged high job strain population had SDNN of 128.3 ± 30.7 ms during the working day and 142.4 ± 28.5 ms during the resting day. We set alpha as 0.05 and the power of the test as 90% (beta = 0.10). With this regard, the calculated sample size was 50 subjects. We anticipated that 20% of Holter ECG data may have been uninterpretable by technical error. Therefore, the estimated sample size was 60 patients.

#### Data analysis

Results were expressed as mean ± SD unless otherwise specified. The numerical data were compared by using paired T-test method. The comparisons of HRV parameters between groups were adjusted according to potential confounding variables. Multivariate analyses were performed using the multiple linear regression. Each variables included in the multiple linear regression were checked for independency using Eta directional measures or Pearson correlation to avoid multicollinearity. Mann–Whitney U test and Wilcoxon signed-rank test were used if data were not normally distributed. We used two-way repeated measures analysis of variance (ANOVA) with pre- and post-call as within-subject factor, and total sleep time quartile as between-subject factor. If the covariance matrix does not appear to have compound symmetry, we analyzed data using generalized estimating equation (GEE). P values of < 0.05 were considered statistically significant. Statistical software package SPSS version 21 was used for analysis.

### Ethics approval and consent to participate

Effects of prolonged working hours on heart rate variability in internal medicine physicians was approved by the ethics committee of the Faculty of Medicine, Chiang Mai University, approval number 268/2564. The investigations were carried out in accordance with the Declaration of Helsinki, including written informed consent of all participants.

## Results

### Baseline characteristics

A total of 60 physicians in the internal medicine training were enrolled in the study. Baseline clinical characteristics and clinical data are shown in Table 1. The mean age of the overall population was 26.0 ± 2.5 years. Majority were females (53.3%). The mean height was 165.5 ± 9.1 cm. The mean body weight was 61.1 ± 14.6 kg. The mean body mass index (BMI) was 22.1 ± 3.6 kg/meters^2^. The mean blood pressure was 116.6 ± 11.4/72.6 ± 8.6 mmHg. The mean heart rate was 78.0±10.7 /min. The mean total sleep time was 238.9 ± 88.3 min. The mean total awake per night was 3 ± 2 times. Of the 60 physicians, 15 (25%) were externs, 10 (17%) were interns, and 35 (48%) were residents.

**Table 1:** Baseline characteristics.

Characteristics	Total number (*N* = 60)
Age	26.0 ± 2.5
Male (%)	28 (46.7)
Height (cm)	165.5 ± 9.1
Bodyweight (kg)	61.1 ± 14.6
BMI (kg/m^2^)	22.1 ± 3.6
SBP (mmHg)	116.6 ± 11.4
DBP (mmHg)	72.6 ± 8.6
Heart rate	78.0 ± 10.7
**Role of job**	
Externs	15(25.0)
Interns	10(16.7)
Residents	35(58.3)
Total sleep time (minutes)	238.9 ± 88.3
Total wake time (minutes)	121 ± 88.3
Total awake per night (times)	3 ± 2

*BMI* body mass index, *DBP* diastolic blood pressure, *SBP* systolic blood pressure.

### The HRV pre-call day and post-call day

Table 2 shows the comparison between pre- and post-call day in overall volunteers. We demonstrated that both time- and frequency-domain HRV parameters did not differ between pre- and post-call day.

**Table 2:** Heart rate variability parameters during 8-h pre-call and post-call.

Heart rate variability parameters	Pre-on-call	Post-on-call	*p* value
Mean heart rate	87.75 ± 9.46	87.88 ± 1.45	0.918
SDNN (ms)	83.82 ± 20.84	86.28 ± 23.13	0.358
SDANN (ms)	63.88 ± 19.66	64.93 ± 19.46	0.717
RMSSD (ms)	27.30 ± 11.17	31.8 ± 29.04	0.219
pNN50 (%)	7.94 ± 10.06	8.03 ± 9.48	0.949
LF	23.92 ± 7.08	23.51 ± 7.59	0.480
HF	12.43 ± 4.26	12.66 ± 5.34	0.620
LF/HF	2.00 ± 0.42	1.94 ± 0.41	0.105
TP	1702.12 ± 901.22	1743.15 ± 1141.63	0.673

*HF* high frequency; *LF* low frequency; *LF/HF* low frequency/high frequency ratio; *pNN50* percentage of successive RR intervals that differ by more than 50 ms; *RMSSD* root mean square of successive RR interval differences; *SDNN* standard deviation of NN intervals; *SDANN-SD* of the average NN intervals; *TP* total power (sum of very low frequency, LF, and HF power) (ms^2^).

### Factors associated with pre- and post-call HRV alterations

We included gender, BMI, total sleep time, and average HR into the multiple linear regression model. The SBP, age, and job role were excluded from the model because of interdependence with BMI and total sleep time. Interestingly, we identified total sleep time during on call as an independent factor associated with SDNN, SDANN, LF, HF, and LF/HF ratio (Table 3).

**Table 3:** Factors associated with pre- and post-call heart rate variability alterations using multiple linear regression analysis.

Factors	SDNN		SDANN		RMSSD		pNN50		LF		HF		LF/HF		TP	
	Beta	P	Beta	P	Beta	P	Beta	P	Beta	P	Beta	P	Beta	P	Beta	P
Gender	0.18	0.207	0.02	0.862	− 0.14	0.383	0.02	0.893	− 0.14	0.339	− 0.05	0.702	0.05	0.713	0.21	0.168
BMI	− 0.18	0.163	− 0.03	0.812	− 0.02	0.918	0.02	0.867	0.02	0.911	− 0.09	0.481	0.14	0.287	− 0.08	0.554
Sleep time	− 0.41	0.002*	− 0.27	0.035*	0.06	0.665	− 0.24	0.084	− 0.30	0.024*	− 0.35	0.008*	0.37	0.006*	− 0.12	0.383
Mean HR	0.22	0.207	0.24	0.053	0.02	0.859	0.19	0.156	0.13	0.297	0.13	0.314	− 0.06	0.633	0.03	0.803

*BMI* body mass index; *HF* high frequency; *HR* heart rate; *LF* low frequency; *LF/HF* low frequency/high frequency ratio; *pNN50* percentage of successive RR intervals that differ by more than 50 ms; *RMSSD* root mean square of successive RR interval differences; *SDNN* standard deviation of NN intervals; *SDANN-SD* of the average NN intervals; *TP* total power (sum of very low frequency, LF, and HF power) (ms^2^).

*P value < 0.05.

In Table 4, two-way repeated measures ANOVA and GEE demonstrated statistically significant interaction between total sleep time and various pre/post call HRV parameters, including SDNN, SDANN, pNN50, LF, HF, and LF/HF ratio. This suggests that different total sleep time could affect pre-/post- call HRV alterations differently. When divided total sleep time into 4 quartiles, there were 18 physicians in the 1st quartile (total sleep time ≤ 180 min) and 15 physicians in the 4th quartile (total sleep time ≥ 307.5 min). Baseline pre-call HRV parameters between 1st and 4th quartile was not statistically different. The physicians in the 1st quartile had significant increase in post-call SDNN, pNN50, HF, and decrease in LF/HF ratio as compared to pre-call values (Table 4). While the physicians in the 4th quartile had completely opposite results, with significant decrease in RMSSD, pNN50, LF, HF, and increase in average HR as compared to pre-call values (Table 4).

**Table 4:** Comparing between pre- and post-call HRV parameters in total sleep time quartile 1 and quartile 4 group.

Heart rate variability	Q1: Sleep time ≤ 180 min (*N* = 18)			Q4: Sleep time ≥ 307.5 min (*N* = 15)			Interaction sleep call *P value
	Pre-call	Post-call	*P* value	Pre-call	Post-call	*P* value	
SDNN	81.11 ± 18.10	95.33 ± 20.72	0.009*	76.33 ± 20.51	70.33 ± 17.16	0.289	0.012*
SDANN	63.94 ± 19.56	74.06 ± 19.15	0.055	58.87 ± 14.24	51.20 ± 13.32	0.177	0.024*
RMSSD	27.39 ± 14.70	29.28 ± 9.21	0.389	25.47 ± 8.69	21.47 ± 6.73	0.096	0.070
pNN50	5.77 ± 4.97	8.68 ± 6.52	0.015*	6.04 ± 5.98	3.31 ± 3.60	0.036*	0.002*
LF	22.44 ± 6.15	23.42 ± 7.10	0.298	23.96 ± 8.22	20.67 ± 6.71	0.001*	0.002*
HF	11.68 ± 3.50	13.52 ± 4.69	0.006*	11.82 ± 4.21	9.52 ± 2.88	0.002*	< 0.001*
LF/HF	1.99 ± 0.44	1.79 ± 0.40	0.002*	2.08 ± 0.43	2.17 ± 0.34	0.171	0.002*
Mean HR	87.39 ± 11.36	86.33 ± 10.22	0.634	86.73 ± 8.56	91.73 ± 11.40	0.039*	0.065
TP	1518.66 ± 633.81	1694.67 ± 826.52	0.071	1215.42 ± 595.89	1248.64 ± 611.87	0.756	0.324

*HR* heart rate, *HF* high frequency, *LF* low frequency, *pNN50* percentage of successive RR intervals that differ by more than 50 ms, Q-quartile, *RMSSD* root mean square of successive RR interval differences, *SDNN* standard deviation of NN intervals, *SDANN-SD* of the average NN interval; *TP* total power (sum of very low frequency, LF, and HF power) (ms^2^).

*P value < 0.05.

### Cardiac arrhythmia

There was no significant arrhythmia observed in our population.

## Discussion

There were 3 main findings from our study: 1) In overall population, there were no statistically significant difference in SDNN, SDANN, RMSSD, pNN50, LF, HF, LF/HF, TP, and average HR between pre-call and post-call; 2) Total sleep time is an independent factor associated with pre and post-call HRV alterations; and 3) Physicians in the internal medicine training who slept ≤ 180 min had different post-call HRV alteration pattern as compared to the physicians who slept ≥ 307.5 min.

Although our population were all physicians in internal medicine training with the same on-call duration (24 + 8 h), they may have slightly different roles depending on the level of training, and varying total sleep time depending on number of new admissions and patient’s condition. Dutheil and colleagues measured HRV during the shift and showed that emergency physicians who worked a 24-h shift had significantly higher physical strain, higher mental fatigue, and lower parasympathetic activity (reflected by lower RMSSD) than ones who worked a 14-h shift^9^. Therefore, differing actual working time among individuals may have obscured the real effects on HRV in our population.

We found total sleep time an independent factor associated with pre and post-call HRV alterations. This is consistent with Bourdillon et al. study which showed a significant impact of sleep deprivation on HRV parameters^14^.

Not only is total sleep time during the shift affecting the HRV, but also the measuring period is crucial. Activities (rest at home, during shift, regular workday), time of the day (day or night), and recording duration (range from 2 min to 24 h) all could affect HRV results. It is important to emphasize that our study is different from most previous studies which measured HRV during < 24-h shift work. Instead, we measured HRV on post-call day after physicians worked a 24-h call and had to continue working on post-call day for additional 8 h (24 + 8).

Among the physicians who were categorized in the 4th quartile of total sleep time (> 307.5 min), the PNN50, LF, HF were significantly decreased and average HR was significantly increased during post-call as compared to pre-call workday. Nevertheless, there was a nonsignificant trend toward increased LF/HF ratio during post-call as compared to pre-call workday. This reflected the greater reduction of HF than LF in those with longer sleep time. LF/HF ratio has been reported to estimate sympathovagal balance. A low LF/HF ratio reflects greater parasympathetic activity relative to sympathetic activity, while a high LF/HF ratio may indicate higher sympathetic activity^5^. HF and pNN50 are closely correlated with parasympathetic nervous system (PNS) activity, and total vagal blockade has been shown to eliminate HF oscillations^15^. LF band could be influenced by both the PNS and sympathetic nervous system(SNS) with various degree depending on activities and conditions^5^. Therefore, the physicians in the long sleep group may have had an increase in SNS activity and PNS withdrawal. This corresponded with the increase in the post-call average HR in this group of physicians. As a result, TP value in 4th quartile sleep group may appear unchanged due to opposite direction of SNS and PNS activity. Our results in the 4th quartile group (more sleep time) resemble the results from several studies among residents, surgeons, and emergency physicians which showed lower LF, lower HF, lower HRV, and higher mean HR during on-call day^6,7,9^. In addition to lower HRV, some studies revealed either higher LF/HF ratio or urine noradrenaline, suggesting predominant SNS activity during on call day^7,9,16^. Pichot et al. reported a decrease in pNN50, LF, HF, and an increase in HR as a parasympathetic withdrawal pattern from cumulated physical fatigue at the workplace^12^.

Interestingly, we found the divergent results among the 1st quartile total sleep time group (≤ 180 min). There was a significant increase in SDNN, pNN50, HF, and decrease in LF/HF, which may have reflected the higher PNS activity^5^. These findings were in contrast to some studies that reported a decrease in HF, LF^7^, and increase in LF/HF ratio^7,9^ among physicians during on call, consistent with a sympathetic dominance. This may be explained by different on-call duration (< 24 h in other studies vs 24 + 8 h in our study), and different recording conditions (during < 24 h on call in other studies vs during extra 8 working hours post 24-h call). Langelotz et al. measured HRV in surgeons during a 24-h shift and demonstrated similar findings to our study that SDNN, RMSSD, pNN50, LF, and HF increased at the end of the 24-h shift^8^. This differing HRV response also seen in one study who reported a reduction in PNS activity (RMSSD) after low-intensity cycling, as opposed to an increase in PNS activity after high-intensity cycling^17^. The interaction between SNS and PNS is complex. While an increase in SNS activity can suppress PNS, higher or overwhelming SNS activity may lead to PNS reactivity or “parasympathetic rebound^5^”. This phenomenon may explain our findings of increased SNS and suppressed PNS activity among more sleep group (> 5 h), as compared to parasympathetic rebound among less sleep group (< 3 h). Total power in less sleep group had an increasing trend post-call day which could be due to PNS activation. Parasympathetic rebound measured by HRV was previously described in long-distance truck drivers and it was associated with sleepiness while driving^18^. We are the first to report HRV in physicians while working an extra 8 h after a 24-h shift work. The results were intriguing especially a PNS rebound among physicians who slept less than 3 h during on call. Whether this finding is a marker of sleepiness or exceeding body’s limits needs further investigations. In medical field, sleepiness in physicians has been shown to associated with increased medical errors^19^. Moreover, night shift works adversely affected mood profiles (vigor-activity, anger-hostility, and fatigue-inertia) among residents^20^.

The strengths of our study are: 1) There were studies on HRV in surgeons, emergency physicians, and mixed specialties residents. We specifically looked at physicians in the internal medicine training; 2) Most studies recorded HRV among physicians during 24-h shift but we are the first to record HRV alteration after the extended 24-h shift. Therefore, providing information regarding effects of prolonged working hour on HRV; 3) We compared HRV between pre- and post-call day from 8 to 4 pm which eliminated the differing influence of physical activities on the circadian changes in heart rate and blood pressure^21,22^, given that the 2 recording periods had similar physical activities; and 4) We recorded HRV during 8-h working period. Therefore, it provided a focused HRV information only during work, and excluded during rest or sleep.

Limitations include but not limited to: 1) We did not measure HRV during 24-h call to support the hypothesis of SNS overactivity that finally lead to parasympathetic rebound; 2) We did not measure short-term clinical effects of HRV alterations, such as emotional distress, physical stress, sleepiness score, cognitive function, or medical error incidence; 3) We did not perform additional autonomic function assessments to support the specific correlation between each HRV parameters and autonomic nervous system activity. However, data on individual HRV metrics corresponding to autonomic nervous system dynamics has been previously described in literatures^5^; 4) Sleep time was not measured directly, hence increasing risk of self-report and recall bias. We attempted to reduce these biases with no demand or desirable characteristics, prospective design, in-person interview, and short length of recall period (immediate post call); and 5) We conducted a pre-post study design that had a single arm and was considered as a self-controlled study. We could not totally exclude the effects from confounding factors which occurred around the same time as the intervention (on call). However, by using the same instruments measured only one day apart in the same working period (8am-4 pm) and environments, the observed post-call HRV changes were mostly attributed to the on-call intervention.

## Conclusions

Effects of prolonged shift work more than 24 h on HRV varied depending on total sleep time during on-call day. When total sleep time was more than 5 h, HRV alteration pattern suggested both increased SNS activity and PNS withdrawal. When total sleep time was less than 3 h, HRV alteration pattern suggested parasympathetic rebound. Whether this PNS rebound detected by HRV is directly associated with short-term or long-term adverse clinical outcomes needs further investigations.

## Data Availability

Effects of prolonged working hours on heart rate variability in internal medicine physicians does not cover data posting in public databases. However, data are available upon request should be sent to bwanwarang@yahoo.com and are subject to approval by the Faculty of Medicine, Chiang Mai University Ethics Committee.
